# Synthesis and Characterization of Polymer-Based Coatings Modified with Bioactive Ceramic and Bovine Serum Albumin

**DOI:** 10.3390/jfb12020021

**Published:** 2021-03-30

**Authors:** Wioletta Florkiewicz, Dagmara Słota, Angelika Placek, Klaudia Pluta, Bożena Tyliszczak, Timothy E. L. Douglas, Agnieszka Sobczak-Kupiec

**Affiliations:** 1 Institute of Materials Science, Faculty of Materials Science and Physics, Cracow University of Technology, 37 Jana Pawła II Av., 31-864 Krakow, Poland; wioletta.florkiewicz@pk.edu.pl (W.F.); bozena.tyliszczak@pk.edu.pl (B.T.); agnieszka.sobczak-kupiec@pk.edu.pl (A.S.-K.); 2 Institute of Inorganic Chemistry and Technology, Cracow University of Technology, 24 Warszawska St., 31-155 Krakow, Poland; wiola1718@gmail.com (A.P.); plutaklaudia@chemia.pk.edu.pl (K.P.); 3 Engineering Department, Lancaster University, Gillow Av., Lancaster LA1 4YW, UK; t.douglas@lancaster.ac.uk; 4 Materials Science Institute, Lancaster University, Gillow Av., Lancaster LA1 4YW, UK

**Keywords:** hydroxyapatite, bovine serum albumin, polyethylene glycol, composite coatings, biomineralization

## Abstract

This study involves the synthesis of hydroxyapatite and describes the preparation and characterization of polymer coatings based on poly(ethylene glycol) diacrylate and poly(ethylene glycol) and modified with bovine serum albumin and hydroxyapatite. Hydroxyapatite was obtained by wet chemical synthesis and characterized by X-ray diffraction and FTIR spectroscopy, and its Ca/P molar ratio was determined (1.69 ± 0.08). The ceramic and bovine serum albumin were used in the preparation of composite materials with the polymeric matrix. The chemical composition of coatings was characterized with FTIR spectroscopy, and their morphology was recorded with SEM imaging. Moreover, the measurements of surface roughness parameters and stereometric research were performed. The prepared coatings were subjected to in vitro studies in simulated body fluid and artificial saliva. Changes in chemical composition and morphology after immersion were examined with FTIR spectroscopy and SEM imaging. Based on the conducted research, it can be stated that applied modifiers promote the biomineralization process. The roughness analysis confirmed prepared materials were characterized by the micrometer-scale topography. The materials morphology and roughness, and the morphology of the newly formed apatite deposit, were dependent on the type of the used modifier, and the artificial fluid used in in vitro studies.

## 1. Introduction

Titanium and its alloys are materials of great interest in biomedical application, chiefly used in the production of implantable devices, such as dental [1] and load-bearing orthopedic implants [2]. However, the implant’s contact with patient tissue may induce bacterial adhesion, causing thrombosis or failed osteointegration [3]. The biofilm consists of various bacterial or even fungal species embedded in an extracellular polysaccharides (PSs) matrix and develops appropriate adhesion to a multitude of different surfaces, including host tissues as well as other bacterial cells, protects them against the host immune defense system, and provides tolerance to antibiotic treatments [4]. Among the known materials used as an antifouling agent, which can reduce the adsorption of biomolecules and attachment of microorganisms, a promising one is polyethylene glycol (PEG) [5]. The excellent biocompatibility of the material is of the crucial need for realizing a therapeutic aim efficiently. Coatings made of PEG suppress platelet adhesion, reducing the risk of thrombus formation, do not show antigenicity and harmful activity toward cells even throughout direct interaction [6].

Design and fabrication of materials with surface adjusted for a particular biological application are essential to the synthesis of biocompatible materials. The induction of the appropriate cellular and tissue response is one of the major challenges that tissue engineering is facing [7]. Taking into consideration the desired characteristics of the various biomaterials, the great interest of the researchers focused on hybrid and composite materials is well-justified. The exploit of the beneficial properties of individual composite components seems to be a good approach to produce multifunctional materials [8]. The combination of polymer with various other organic and inorganic substances can enhance cellular interaction, integration of material with the host bone, and tune degradation kinetics of material [8,9,10]. Much effort has been dedicated to developing bioactive materials stimulating a biological response leading to the formation of host tissue-implant connection [11]. Among synthetic and natural inorganic ceramic materials, hydroxyapatite (HA) has been considered a proper candidate for enhancing the regeneration-supportive properties of scaffold materials. The mineral composition likeness of HA and bone and tooth enamel makes HA a suitable biomaterial for bone substitutes [12,13]. Thus, HA is employed as a filler in enamel [14] and jaw bone defects [15]. However, despite its good biological properties, the brittle nature of HA restricts its application in load-bearing implant production [16]. Taking into account difficulties associated with poor mechanical properties of bioceramics and lack of chemical bonding between the inert metallic implant and host tissues as well, the application of surface-modified implants seems to be a solution worthy of considering. Therefore, HA is usually used as a coating on metallic implants [17] or applied as a filler in polymer matrices to prepare composites [18]. Moreover, HA nanometric structures can be used as drug delivery systems with controlled active substances releasing [19] and as a contrast agent for magnetic resonance imaging [20].

Other well-known substances providing suitable conditions for the growth of the cells are protein-derived biomaterials, including collagen [21], fibronectin [22], albumin [23], and gelatin [24]. Albumin is the most abundant human plasma protein, constituting over 50% of the total protein present in the bloodstream, and it is composed of low content of tryptophan and methionine and a high content of cystine and charged amino acids, aspartic and glutamic acids, lysine, and arginine [25]. Bovine serum albumin (BSA) or Fraction V, due to its biocompatibility and non-toxicity, is widely used as a drug delivery system [26] and for tissue engineering applications [26]. BSA is acquired by purification of blood obtained as a by-product of the cattle industry, thus provides an inexpensive substance for potential application in the biomedical area [27]. BSA has been used in molecular biology as a constituent of coatings blocking surface sites from non-specific adsorption, which found application in a microfluidic polymerase chain reaction (PCR) [28]. BSA, due to its anti-adsorption properties, was also tested from the viewpoint of bactericidal activity. Experiments show that BSA reduced bacterial colonization of the *Staphylococcus epidermidis* strain on titanium surfaces in vitro [29,30].

This work is devoted to the fabrication and in vitro characterization of composite material coatings consisting of hydroxyapatite, BSA, and PEG/PEGDA polymeric matrix obtained via the photopolymerization route, giving insight into coatings composition-dependent biomineralization processes in simulated body fluid (SBF) and artificial saliva. To the best of our knowledge, there are no studies dealing with the method of preparation, physicochemical properties, and bioactivity of such obtained materials.

## 2. Materials and Methods

### 2.1. Reagents

Polyethylene glycol of average molecular weight 8000 g/mol was purchased from Acros Organics. Bovine serum albumin (BSA; >98% pure), poly(ethylene glycol) diacrylate (PEGDA, average molecular weight 700 g/mol) used as a crosslinking agent, and 2-hydroxy-2-methylpropiophenone (Darocur 1173) employed as a photoinitiator were acquired from Sigma-Aldrich. The following were used to composite SBF: sodium chloride, sodium bicarbonate, potassium chloride, potassium phosphate, potassium phosphate dibasic trihydrate, magnesium dichloride dihydrate, hydrogen chloride, calcium chloride, sodium sulfate and Tris buffer solution. For the preparation of artificial saliva, sodium chloride, potassium chloride, calcium chloride, sodium dihydrogen phosphate monohydrate, sodium sulfide nonahydrate and urea were used. All these reagents were purchased from Sigma-Aldrich.

### 2.2. Preparation of Hydroxyapatite

Hydroxyapatite powder was obtained via the wet precipitation method in the reaction of Ca(OH)_2_ and H_3_PO_4_. To obtain HA slurry, 500 mL of 0.3 M H_3_PO_4_ aqueous solution was added dropwise to 500 mL of 0.5 M aqueous solution of Ca(OH)_2_ under constant intense stirring. The pH of the mixture was kept at ~11 by using an aqueous ammonia solution (25%). When the addition was accomplished, the reaction mixture was aged for 24 h. The precipitate was filtered through the paper filter, washed with distilled water, dried in a laboratory drier at 104 °C for 4 h, and calcined for 1 h in a chamber furnace in an air atmosphere at 750 °C.

### 2.3. Preparation of Coatings

To obtain mixtures intended for coatings material preparation, 20% solution of poly(ethylene glycol) and 2% solution of BSA was prepared. The appropriate amounts of such-prepared solutions, as well as HA powder, were utilized to obtain mixtures used in composite coating preparation. As a crosslinking agent and photoinitiator, poly(ethylene glycol), diacrylate and 2-hydroxy-2-methylpropiophenone (Sigma-Aldrich) were used respectively. A detailed description of the composition of mixtures used for coatings preparation is shown in Table 1.

100 µL of each mixture were pipette onto Ti-6Al-4V plates (2 cm × 2.5 cm). Plates were acid-etched in 1% HF solution for 10 min, immersed in ethanol (96%) and distilled water, subsequently cleaned by sonication in acetone and ethanol, and finally rinsed with double distilled water. Photopolymerization of coatings was performed with UV lamp EMITA VP 60 (180 W) for 3 min. Subsequently, such-prepared coated plates were incubated for 21 days at 37 °C in 30 mL of simulated body fluid (SBF) and artificial saliva.

### 2.4. XRD Analysis

To perform structural characterization of HA, X-ray diffraction analysis with the use of XRD diffractometer (Pananalytical X’Pert, Worcestershire, UK) with Cu Kα radiation (λ = 0.15418 nm), equipped with graphite monochromator PW 1752/00 operating at 40 kV and 30 mA in 2θ range of 25°–55° was employed.

### 2.5. FT-IR Analysis

To identify the functional groups in the HA sample, as well as perform analysis of coatings before and after incubation, infrared spectroscopy was used with a FT-IR spectrometer (Thermo Scientific, Nicolet iS5 FTIR, Waltham, MA, USA) equipped with iD7 ATR accessory operated at room conditions in the range of 4000 cm^−1^–400 cm^−1^.

### 2.6. Determination of Calcium and Phosphorus Content in HA

Calcium and phosphorus content in HA powder was examined utilizing complexometric titration and spectrophotometric methods, respectively. Based on the determined calcium and phosphorus content in apatite, the molar ratio of Ca/P in the material was calculated. To determine Ca content, two samples of HA powder of 0.1 g were prepared by separately dissolving in 10 mL of HNO_3_ at a concentration of 3 mol/L and boiling for 10 min. Subsequently, 20 mL of redistilled water was added to mixtures and boiled for another 5 min. The cooling solutions were transferred quantitatively to 100 mL volumetric flasks, and 6.25 mL of Bi(NO_3_)_3_ at a concentration of 0.4 mol/L was added. Finally, the flask was makeup with distilled water. After 3 min, solutions were filtered through a double hard qualitative filter. 25 mL of filtrate, 25 mL of redistilled water, and 3 mL of a 25% solution of triethanolamine (TEA) were placed and mixed in a conical flask, neutralized with a 20% solution of KOH to a pH between 5 and 7, and another 10 mL of 20% potassium hydroxide and a pinch of the indicator were added and mixed. The such-obtained mixtures were titrated with a 0.02 mol/L standard solution of edetate disodium. Both samples were analyzed in triplicate.

For the sake of phosphorus content determination in HA, two samples of obtained 0.02 g were treated with 65% HNO_3_ and 36% HCl at a volume ratio of 3:1 and boiled until the color of the vapor changes from orange to colorless. Then, 40 mL of redistilled water was added and boiled for another 5 min. Subsequently, solutions were transferred quantitatively to 100 mL volumetric flasks and makeup with distilled water, mixed, and filtered through a medium qualitative filter. Finally, 10 mL of filtrate and 20 mL of D solution (solution of molybdenum–vanadium complex) were placed in a 50 mL volumetric flask and makeup with redistilled water. After 15 min absorbance of each mixture was measured. The measurements of absorbance were carried out at 430 nm with a UV-vis Thermo Scientific Evolution spectrophotometer.

### 2.7. Morphology Analysis

The studies of morphology of samples were taken with scanning electron microscope (Zeiss, Ultra Plus, Oberkochen, Germany) equipped with EDS microanalysis system Quantax 400 V (Bruker) with an ultra-fast detector with 127 eV energy resolution. The SEM analysis was performed for the sake of visualization of hydroxyapatite and coatings morphology, as well as recording potential deposits formed on the surface of the samples.

### 2.8. Measurements of Surface Roughness Parameters and Stereometric Research

The roughness analysis of the tested samples was carried out using a Form Talysurf Series profilometer (Rank Taylor Hobson Ltd., Form Talysurf Series 2, Leicester, UK). A phase correction band filter (Gaussian filter) was applied for the measurements. The value of the elementary segment was lr = 0.8 mm, and the measurement section ln = 4.0 mm. Roughness measurements were repeated five times on each surface.

## 3. Results

### 3.1. XRD Analysis

**Figure 1:**
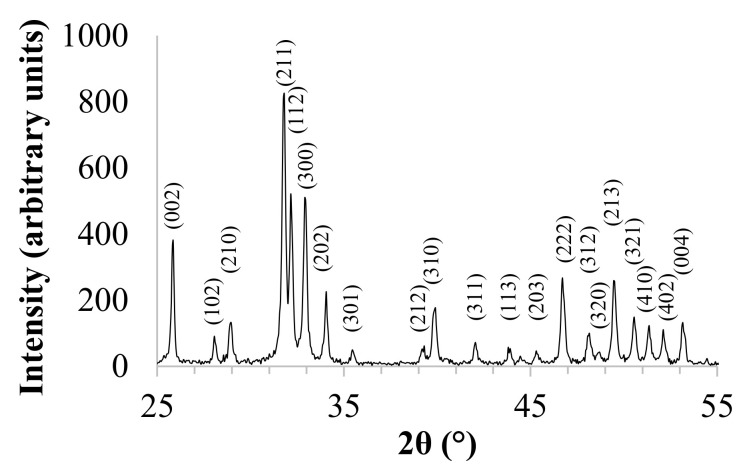
XRD pattern of hydroxyapatite (HA).

XRD pattern is shown in Figure 1. In accordance with International Center for Diffraction Data (No. 9-432), XRD reflections were assigned to the hexagonal structure of HA, and the calculated lattice parameters were a = 9.4172 Å and c = 6.8799 Å. The peak with the highest intensity is observed at 31.74 of two theta, which corresponds to the main peak of the hexagonal HA structure. The fraction of crystalline phase (X_c_) of the powder was calculated in accordance with the following Equation (1):

(1) $$X_{c}=100\;\times \;\frac{I_{300}V_{112/300}}{I_{300}}$$

where I_300_ is the intensity of (300) diffraction peak and V_112/300_ is the intensity of the hollow between (112) and (300) diffraction peaks [31], and the calculated X_c_ was 88%. Furthermore, for HA, the (211) and (121) crystallographic planes form a doublet, which is typical of stoichiometric and highly crystalline hydroxyapatite.

The ab-plane orientation degree of HA was calculated on the basis of the intensities of the basis of the intensities of the 300, 211 and 002 reflections, in accordance with Equation (2):

(2) $$\mathrm{Orientation}\;\mathrm{degree}\;\mathrm{of}\;\mathrm{the}\;a,b-\mathrm{plane}=100\%\;\times \;\left[\frac{I_{300}}{I_{300}+I_{211}+I_{002}}\right]$$

where I_300_ is the intensity of the (300) diffraction peak, I_211_ is the intensity of the (211) diffraction reflection, and I_002_ is the intensity of the (002) diffraction peak. The calculated a,b-plane orientation degree of HA was 29%.

### 3.2. Determination of Calcium and Phosphorus Content in HA

The determined calcium and phosphorus contents in HA powder, as well as the calculated Ca/P molar ratio, are presented in Table 2. The calculated Ca/P molar ratio is slightly different from the stoichiometric value, which can be caused by partial substitution of phosphate ions by carbonate groups, which is inevitable during the synthesis under the atmospheric condition.

### 3.3. FT-IR Analysis

#### 3.3.1. Hydroxyapatite

**Figure 2:**
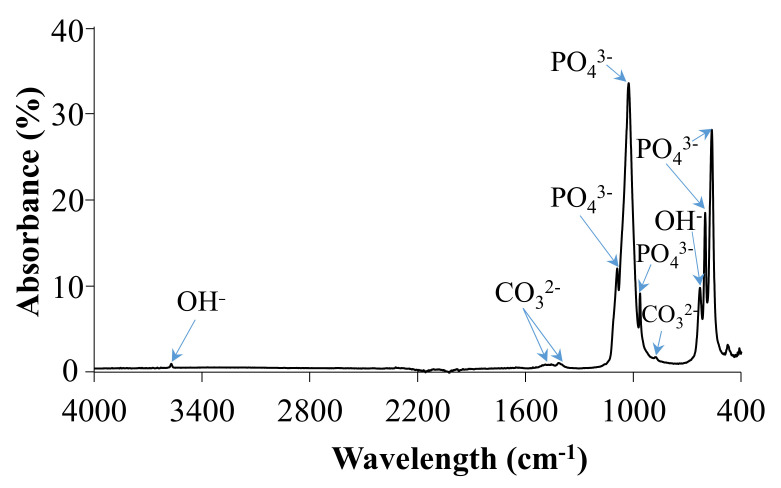
FTIR spectrum of HA.

IR spectrum of HA powder is shown in Figure 2 and summarized in Table 3. The bands observed at 879 cm^−1^, 1410 cm^−1^ and 1455 cm^−1^ in the IR spectrum were attributed to ν_2_ bending mode and ν_3_ stretching modes of carbonate ions. IR spectrum of ideally stoichiometric HA should not reveal any carbonate modes; however, their presence may be due to atmospheric adsorption after the synthesis. In the 1000–1100 cm^−1^ region, bands centered at 1025 cm^−1^ and 1090 cm^−1^ were attributed to the ν_3_ triply degenerate asymmetric stretching mode of PO_4_^3−^.

Furthermore, two distinctive peaks observed at 630 cm^−1^ were assigned to δ in-plane vibration of OH^−^ and ν_s_ stretching mode of the structural OH anion, respectively. The band at 963 cm^−1^ was attributed to the ν_4_ bending vibration of PO_4_^3−^. Two sharp and intensive bands located at 601 cm^−1^ and 563 cm^−1^ correspond to ν_4_ triply degenerate bending mode of PO_4_^3−^, while the band at 471 cm^-1^ represents ν_2_ doubly degenerate bending mode of phosphate ions.

#### 3.3.2. Coatings before Incubation

**Figure 3:**
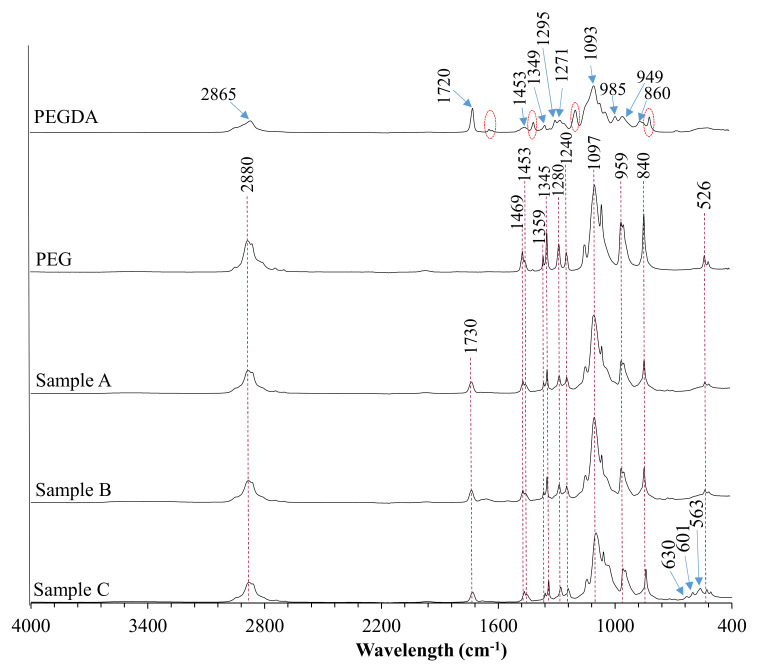
FTIR spectra of poly(ethylene glycol) diacrylate (PEGDA), polyethylene glycol (PEG), and coatings.

In order to determine the chemical composition of the prepared coatings, as well as to verify complete polymerization of all specimens, they were investigated with the use of diamond crystal ATR FT-IR spectroscopy (Figure 3).

The absorption band of the PEGDA sample at 2865 cm^−1^ was attributed to the C–H stretching vibrations, whereas the band centered at 860 cm^−1^ represents C–H bending mode. The peak at 1720 cm^−1^ was observed due to the frequency of the C=O stretching. It is worth noticing that materials crosslinking resulting in the shifting of this peak from 1720 cm^−1^ to 1730 cm^−1^. Absorption bands indicated with the red dashed line were assigned to C=C symmetric stretching and were not recorded in the crosslinked materials. The peak centered at 1453 cm^−1^ was assigned to symmetric bending of the CH_2_ group. Bands located at 1349 cm^−1^ and 1295 cm^−1^ were attributed to C–O asymmetric bending, whereas peaks at 1093 cm^−1^ and 950 cm^−1^ correspond to C–O–C stretching. The PEG spectrum showed characteristic bands of specific functional groups, such as the broad bands appearing at 2880 cm^−1^ assigned to C–H symmetric stretching vibrations and the band at 1097 cm^−1^ assigned to the–C–O–C group. The peak at 1469 cm^−1^ corresponds to the in-plane scissoring of the CH_2_ group. Bands located at 1359 cm^−1^ and 1345 cm^−1^ represent in-plane O–H deformation, whereas bands centered at 1280 cm^−1^, 959 cm^−1^, 840 cm^−1^, and 526 cm^−1^ were attributed to C–C skeletal stretching, and the peak at 1240 cm^−1^ represents C–O–C stretching mode. Moreover, some additional peaks related to BSA and HA presence were observed at 1654 cm^−1^ and 550 cm^−1^ to 650 cm^−1^ wavelength region, respectively.

#### 3.3.3. Coatings after Incubation

**Figure 4:**
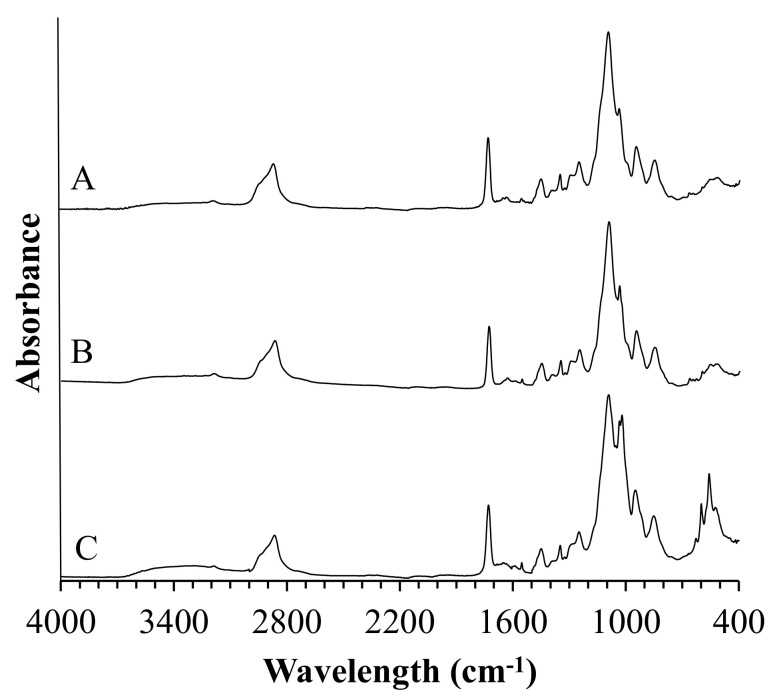
FTIR spectra of coatings samples after incubation in simulated body fluid (SBF).

**Figure 5:**
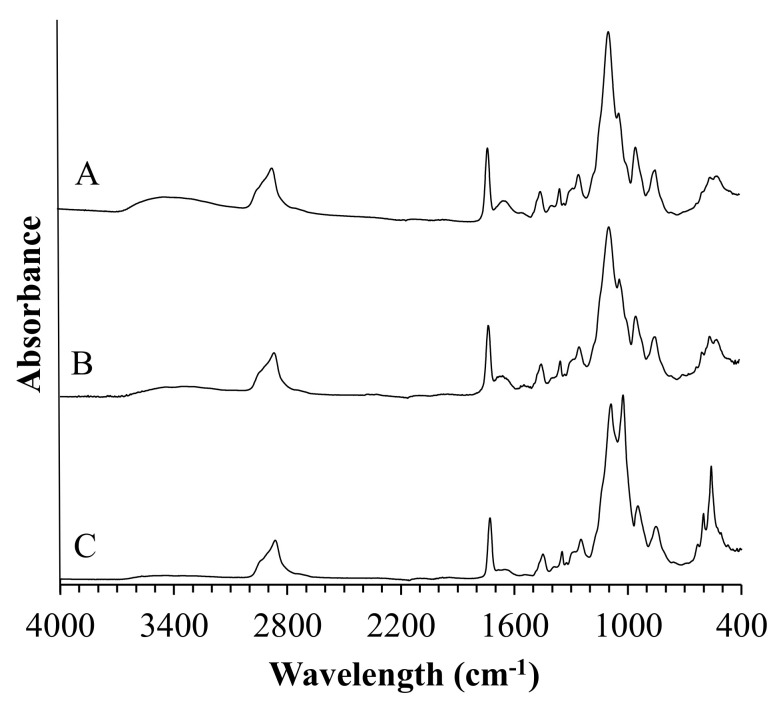
FTIR spectra of coatings samples after incubation in artificial saliva.

FTIR technique can be a useful tool for gaining more insight into the process of biomineralization of materials during incubation. FTIR spectra of composite coatings after immersion in SBF and artificial saliva are shown in Figure 4 and Figure 5, respectively.

FTIR analysis of composite coatings after incubation revealed the presence of new vibrational modes in the wavelength range from 500 cm^−1^ to 650 cm^−1^ and 1500 cm^−1^ and 1600 cm^−1^. Moreover, it was observed that composites incubated in artificial saliva exhibited a more narrow and sharp distribution of the newly formed FTIR bands as compared to spectra registered for specimens incubated in SBF.

### 3.4. Morphology Analysis

#### 3.4.1. Hydroxyapatite and Titanium Alloy Plates

**Figure 6:**
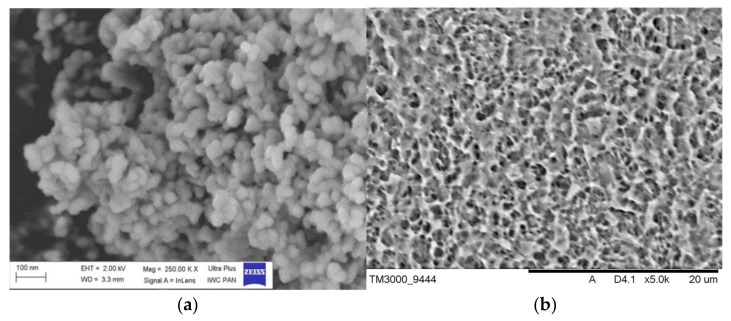
SEM image of the obtained HA powder (**a**) and Ti alloy after acid etching (**b**).

Figure 6 shows the SEM images of the as-prepared HA (Figure 6a) and acid-etched titanium alloy plate (Figure 6b). The SEM microphotography showed that the formed hydroxyapatite particles were highly agglomerated. The agglomeration of the particles might be because of Ostwald ripening [32]. The SEM image shows the spherical-shaped particles of about 50 nm and clumped distributions. Acid etching of Ti alloy plates results in a homogeneous distribution of ridges and valleys of various dimensions and irregularities throughout the surface.

#### 3.4.2. Composite Coatings before Incubation

**Figure 7:**
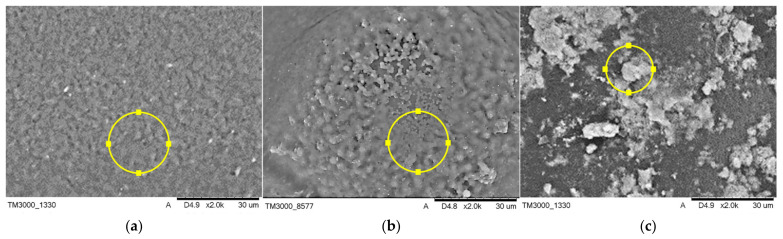
SEM micrographs of coatings composed of PEG (**a**), modified with bovine serum albumin (BSA) (**b**), and HA (**c**) before incubation.

Morphology and elemental composition of the tested composite coatings before incubation in artificial body fluids are presented in Figure 7, and the compositional analysis is presented in Table 4.

The morphology of the tested composite materials is comparable, while the visible pores of various shapes and sizes are present all over the samples. EDS elemental analysis was performed in order to investigate the elemental composition of the materials. C and O originating from the polymeric matrices were found to be present in all specimens. The point analysis of the elemental composition also confirmed the presence of HA in sample C.

#### 3.4.3. Composite Coatings after Incubation

**Figure 8:**
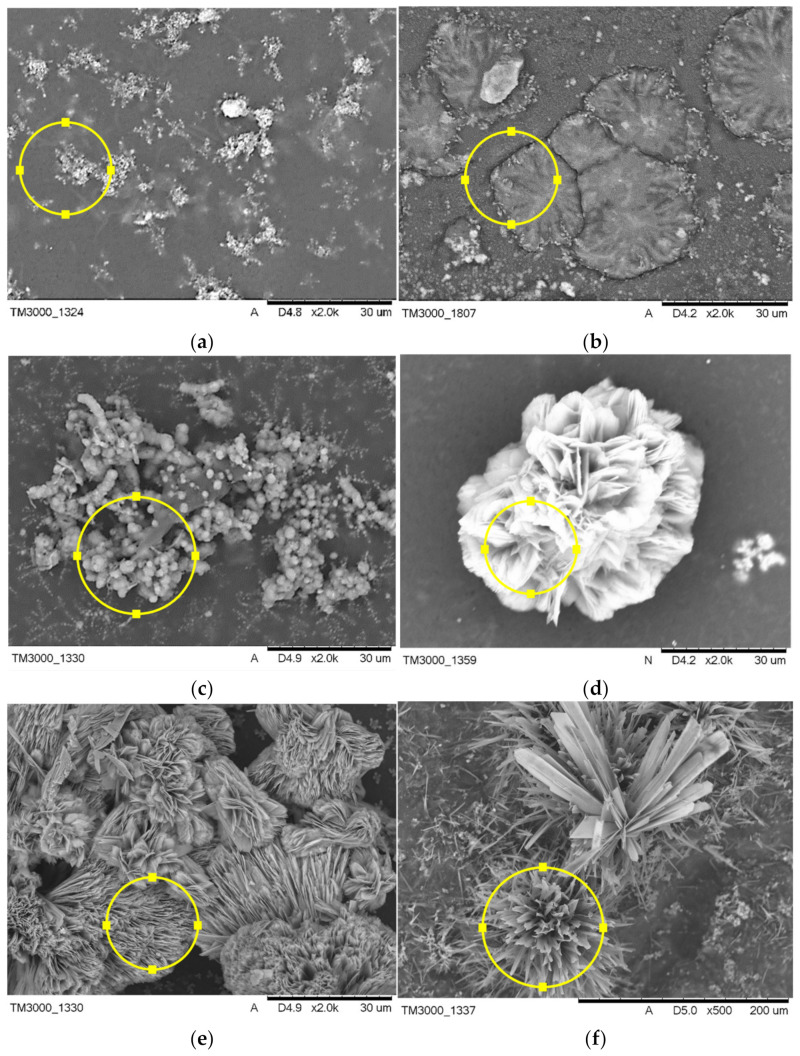
SEM micrographs of structures formed on coatings composed of PEG, modified with BSA, and HA incubated in SBF (**a**–**c**) and artificial saliva (**d**–**f**).

The surface morphology of composite coatings after incubation in SBF and artificial saliva is shown in Figure 8, and the compositional analysis is presented in Table 5. The coatings modified with HA and BSA and polymeric coating showed signs of apatite formation after 21 days of incubation in both artificial body fluids. Comparatively, there were significant differences in morphology of the formed apatite layer on the composite material, dependent on their composition, implying the crucial role of modifiers in the biomineralization process. Six distinct microstructures of apatite of different crystal sizes were observed on the surfaces of specimens by SEM, as shown in Figure 8. Incubation of sample A in SBF results in the formation of evenly distributed fine-grained clusters of apatite, whereas immersion in artificial saliva leads to flower-like morphology of apatite. The addition of BSA to the polymeric matrix causes the formation of arrays of the long thin needle and comparatively large plate-like clusters in SBF and artificial saliva, respectively. The use of HA as a modifier favors the formation of sphere-like phosphates with a single unit of approximately 2 µm diameter during incubation in SBF, whereas immersion in artificial saliva promotes the formation of thick column-like structures.

### 3.5. Stereometric and Surface Roughness Analysis

**Figure 9:**
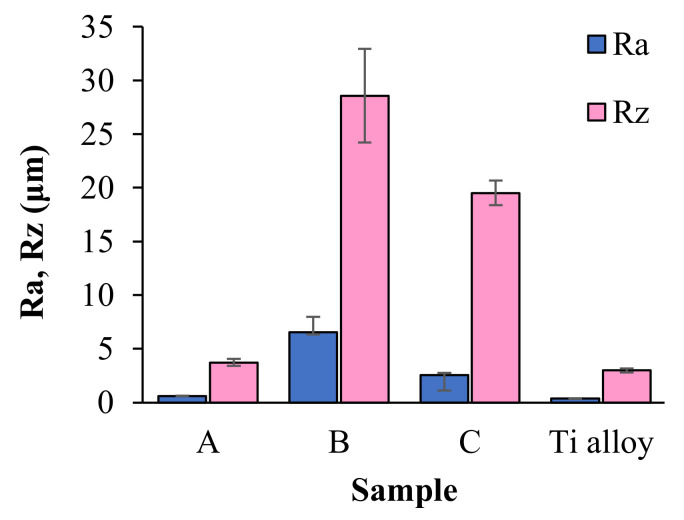
The values of *Ra* and *Rz* parameters recorded for the titanium alloy plate and the coatings samples.

Figure 9 shows roughness parameters *Ra* (arithmetic mean deviation of the assessed profile) and *Rz* (maximum height of the profile) of coatings of the proposed compositions. The *Ra* and *Rz* parameters are in the wide range from 0.60 ± 0.04 μm to 6.56 ± 1.41 μm and 3.71 ± 0.32 μm to 28.56 ± 4.36 μm, respectively. It can, therefore, be seen that the addition of BSA and HA does significantly affect the surface roughness. The samples containing BSA gave the highest values of both roughness parameters, whereas the lowest values among coatings were recorded for sample A. In comparison with coatings samples, *Ra* and *Rz* parameters of acid-etched Titanium alloy are in a lower (0.36 ± 0.03 μm and 2.99 ± 0.20 μm, respectively).

**Figure 10:**
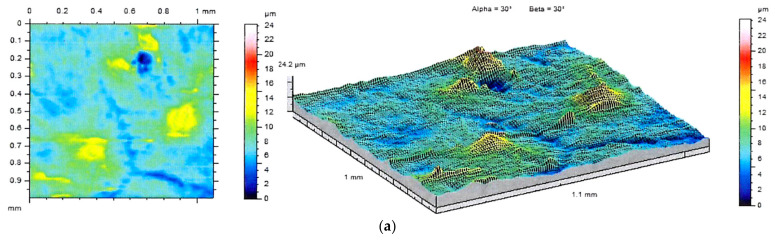

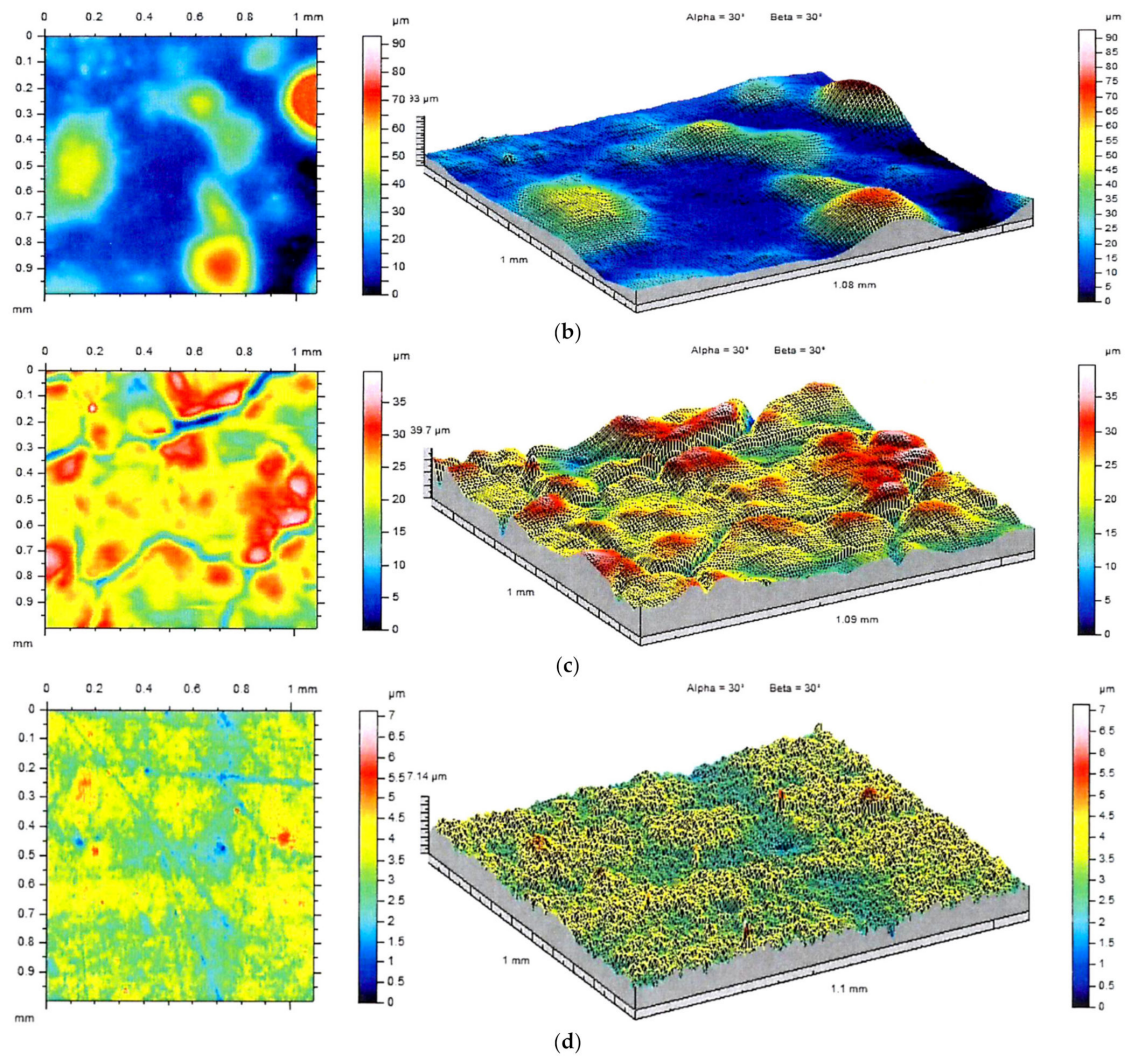
2D images and topographic views of the samples A (**a**), B (**b**) and C (**c**)**,** and titanium substrate (**d**) surface.

The differences between analyzed surfaces were also evident in the images of stereometric structure (Figure 10). It can be seen that the addition of BSA results in the formation of individual irregularities distributed on the sample surface, whereas the PEG-containing specimens are relatively smooth. The irregularities visualized in the isometric views can also be observed in the SEM images.

## 4. Discussion

X-ray diffraction phase composition analysis of the ceramic powder showed a high crystallinity of the powder obtained via the chemical precipitation, and the only phase present in the specimen was found to be hydroxyapatite. Moreover, on the basis of the XRD pattern, the ab-plane orientation degree was calculated. Zhuang et al. [33] proved the orientation degree is strongly associated with surface wettability and can affect the adhesion efficiency of MC3T3-E1 cells to materials. FTIR spectrum of the prepared material showed the characteristic bands of HA. The analysis also revealed the presence of bands assigned to carbonate ions located at 1455 cm^−1^ and 1410 cm^−1,^ characteristic for B-type apatite and peak at 879 cm^−1^ pertaining to A-type apatite [34]. Determination of Ca and P contests, and the Ca/P molar ratio calculated on their basis, suggest that prepared ceramic material met the requirements of ISO13779-1:2008 standard, which states that the Ca/P molar ratio should be in the range 1.5–2.0 [35].

The FTIR analysis of PEGDA and composite coatings confirmed complete polymerization of the crosslinking agent in all specimens, which is stated based on the disappearance of bands assigned to C=C symmetric stretching indicated with the red dashed lines in the non-cross-linked PEGDA. Moreover, the shifting of the peak located originally at 1720 cm^−1^ (C=O stretching) at the spectrum of non-cross-linked PEGDA to 1730 cm^−1^ after UV light treatment of specimens was observed, which is due to the saturation of double bonds adjacent to the carbonyl group [36].

FTIR analysis of composite coatings after 21 days of immersion in artificial body fluids revealed the formation of new bands at the 500 cm^−1^ to 700 cm^−1^ wavelength region for specimens incubated in simulated body fluid and artificial saliva as well. The absorption bands at this range may suggest the formation of apatite deposits on the materials during incubation, which was confirmed with SEM-EDS analysis [37]. FTIR spectra of composites after incubation in artificial saliva exhibited a more narrow and sharp distribution of the newly formed bands as compared to spectra registered for SBF-incubated samples, indicating probably slightly higher crystallinity of calcium phosphate layer. However, unambiguously determination of the type of the formed deposit is not possible due to the complexity of the FTIR spectra of composites. SEM imaging revealed the formation of apatite deposits of various morphology on all of the coatings. This implies the modifier-dependent as well as solution type-dependent crystallization of apatite. Following the literature, BSA may inhibit or promote HA nucleation. Experiments performed in bufferless simulated inorganic plasma (HBSS) revealed that biomineralization is promoted when albumin is pre-adsorbed and inhibited when it is dissolved in HBSS. This effect is caused by the presence of charged residues that can bind to phosphate and calcium ions, inhibiting the formation of apatite deposits. The aspartic and glutamic acid residues could bind to the calcium site, whereas lysine and arginine could bind to phosphate groups at the early stage of mineralization processes [38]. The SEM imaging showed the formation of larger individual crystals on polymeric samples incubated in artificial saliva, whereas immersion in SBF results in the formation of fine-grained clusters of apatite. Moreover, our experiments clearly indicate that the addition of BSA to polymeric matrix promotes mineralization of calcium deposits on composite surfaces in both used artificial fluids leading to the formation of about 60 µm clusters. The effect of various amino acids on crystallinity and morphology of HA was investigated by Matsumoto et al. [39]. The experiment revealed that some of the amino acids, including glycine, serine, and glutamic acid, can affect the morphology of the synthesized HA, leading to the formation of plate-like apatite. Numerous reports indicate the morphology of the biomineralized apatite is dependent on the investigated material surface [40]. Here we report that type of solution also affects the structure of the formed deposits. As it is shown in SEM images of HA-containing composites incubated in SBF and artificial saliva, differences in the formed apatite deposit morphology are indisputable. In the case of artificial saliva, the column-like shape of crystals and their ordered arrangement suggest that crystallization of apatite proceeds along with some preferred orientation, probably the fast-growing directions of HA points out of the sample. Moreover, the observed structures are of the greatest size among other recorded deposits. The more efficient crystallization, in this case, can be caused by the lower pH of the artificial saliva in comparison with PBS, which leads to the faster dissolution of hydroxyapatite present in the coatings, local supersaturation of liquid, leading to faster nucleation and crystal growth. A similar tendency was not observed for the sample immersed in SBF solution. Here, the formed apatite structures were of spherical shape.

The performed measurements of surface roughness parameters and stereometric research revealed the micrometer-scale topography of the prepared materials with roughness parameters *Ra* and *Rz* dependent on materials composition. The bone integration with the implant surface is reliant on the topography of biomaterial [41,42]. In accordance with studies presented by Wennerberg et al. smooth (*Ra* < 0.5 μm) and minimally rough (*Ra* < 0.5–1 μm) surfaces revealed less strong bone responses than rougher surfaces, whereas surfaces of moderately roughness (*Ra* > 1–2 μm) showed stronger bone responses than rough (*Ra* > 2 μm) in some studies [43]. Based on this statement, it can be assumed that samples A and C are the most promising materials from the viewpoint of their potential biomedical application.

Summarizing the tested composite coatings based on synthetic polymers, proteins of natural origin, and hydroxyapatite due to their bioactivity can be promising materials for utilization in the field of medicine and dentistry.

## 5. Conclusions

A novel phosphate–ceramic coating was prepared for potential biomedical applications. The in vitro bioactivity tests of the prepared coatings in SBF and artificial saliva were carried out with an analysis of the changes of the chemical composition of coatings during incubation and imaging of the morphology of the formed apatite deposit. The results of this study can be summarized as follows:

1. The HA powder with a Ca/P molar ratio of 1.69 ± 0.08 and the fraction of the crystalline phase of 88% was obtained. The material met the ISO standard requirement;
2. Modification of PEG/PEGDA coatings with BSA and HA results in the promotion of the biomineralization process;
3. The morphology of the formed apatite structures is dependent on the type of the used modifier, as well as on the artificial fluid used in in vitro studies;
4. Based on the obtained FTIR spectra, it can be concluded that samples incubated in artificial saliva are covered with more crystalline newly-formed apatite deposits;
5. Morphology of samples incubated in artificial saliva suggests direction crystallization of the formed apatite structures.

## Figures and Tables

**Table 1:** Composite materials composition.

Sample Symbol	PEG (mL)	BSA (mL)	PEGDA (mL)	Photoinitiator (µL)	Hydroxyapatite (g)
A	18	0	2	40	0
B	17	1			0
C	18	0			2

**Table 2:** Calcium and phosphorus content and Ca/P molar ratio in HA (mean ± SD).

Ca Content (wt %)	P Content (wt %)	Ca/P Molar Ratio
41.06 ± 0.33	18.80 ± 0.48	1.69 ± 0.08

**Table 3:** IR band assignment of HA.

Wavenumber (cm^−1^)	Peak Assignment
3571	Band corresponding to H_2_O absorption
1455	ν_3_ stretching mode of CO_3_^2−^
1410	ν_3_ carbonate ions
1090	ν_3_ asymmetric stretching mode of P–O
1025	ν_3_ asymmetric stretching mode of P–O
962	ν_1_ symmetric stretching mode of P–O
879	ν_2_ bending mode of carbonate ions
630	δ in-plane vibration of OH^−^
601	ν_4_ triply degenerate bending mode of PO_4_^3−^ (O–P–O)
563	ν_4_ triply degenerate bending mode of PO_4_^3−^ (O–P–O)

**Table 4:** Elemental microanalysis on points indicated in Figure 7.

Sample	Mass Percentage (wt %)
A	C: 36.6, O: 34.8, Au: 15.5
B	C: 59.9, O: 37.5, Au: 2.6
C	C: 37.1, O: 34.7, Ca:18.2, P: 7.7, Au: 2.3

**Table 5:** Elemental microanalysis on points indicated in Figure 8.

Sample	Mass Percentage (wt %)
a	C: 44.3, O: 40.3, Ca: 8.7, P: 4.9, Na: 1.0, Cl: 0.8
b	C: 47.1, O: 39.9, Ca: 7.2, P: 4.0, Na: 2.4, Cl: 2.3
c	C: 38.0, Ca: 23.4, C 18.2, P: 14.1, Cl: 2.8, Mg: 1.4, K: 1.2, Na: 0.9
d	O: 48.4, Ca: 28.4, P: 12.6, C: 10.6
e	O: 50.4, Ca: 26.7, P: 12.2, C: 9.6, Na: 0.6, Cl: 0.5
f	O: 46.3, C: 26.2, Ca: 19.6, P: 7.8, Cl: 0.1

## Data Availability

All data presented and analyzed in this study are included in this published article. Raw data may be provided by the authors upon a justified request.

## References

[1] Romanos G.E., Fischer G.A., Delgado-ruiz R. (2021). Titanium wear of dental implants from placement, under loading and maintenance protocols. Int. J. Mol. Sci..

[2] Yoshimitsu Okazaki J.M. (2021). Mechanical Performance of Artificial Hip Stems Manufactured. Materials.

[3] Dong H., Liu H., Zhou N., Li Q., Yang G., Chen L., Mou Y. (2020). Surface modified techniques and emerging functional coating of dental implants. Coatings.

[4] Muhammad M.H., Idris A.L., Fan X., Guo Y., Yu Y., Jin X., Qiu J., Guan X., Huang T. (2020). Beyond Risk: Bacterial Biofilms and Their Regulating Approaches. Front. Microbiol..

[5] Zeng G., Ogaki R., Meyer R.L. (2015). Non-proteinaceous bacterial adhesins challenge the antifouling properties of polymer brush coatings. Acta Biomater..

[6] Xu L., Bauer J.W., Siedlecki C.A. (2014). Proteins, Platelets, and Blood Coagulation at Biomaterial Interfaces. Colloids Surf B Biointerfaces.

[7] Chandra P.K., Soker S., Atala A. (2020). Tissue engineering: Current status and future perspectives. Principles of Tissue Engineering.

[8] Ali A., Andriyana A. (2020). Properties of multifunctional composite materials based on nanomaterials: A review. RSC Adv..

[9] Senra M.R., Marques M.D. (2020). Synthetic Polymeric Materials for Bone Replacement. J. Compos. Sci..

[10] Zimina A., Senatov F., Choudhary R., Kolesnikov E., Anisimova N., Kiselevskiy M., Orlova P., Strukova N., Generalova M., Manskikh V. (2020). Biocompatibility and physico-chemical properties of highly porous PLA/HA scaffolds for bone reconstruction. Polymers.

[11] Shi H., Zhou Z., Li W., Fan Y., Li Z., Wei J. (2021). Hydroxyapatite Based Materials for Bone Tissue Engineering: A Brief and Comprehensive Introduction. Crystals.

[12] Bordea I.R., Candrea S., Alexescu G.T., Bran S., Băciuț M., Băciuț G., Lucaciu O., Dinu C.M., Todea D.A. (2020). Nano-hydroxyapatite use in dentistry: A systematic review. Drug Metab. Rev..

[13] Bal Z., Kaito T., Korkusuz F., Yoshikawa H. (2020). Bone regeneration with hydroxyapatite-based biomaterials. Emergent Mater..

[14] Onuma K., Iijima M. (2017). Artificial enamel induced by phase transformation of amorphous nanoparticles. Sci. Rep..

[15] Mamun S., Akhter M., Molla M.R. (2009). Bone Grafts in Jaw Cysts- Hydroxyapatite & Allogenic Bone—A Comparative Study. Bangabandhu Sheikh Mujib Med. Univ. J..

[16] Kattimani V.S., Kondaka S., Lingamaneni K.P. (2016). Hydroxyapatite—Past, Present, and Future in Bone Regeneration. Bone Tissue Regen. Insights.

[17] Yu H.N., Hsu H.C., Wu S.C., Hsu C.W., Hsu S.K., Ho W.F. (2020). Characterization of nano-scale hydroxyapatite coating synthesized from eggshells through hydrothermal reaction on commercially pure titanium. Coatings.

[18] Pluta K., Florkiewicz W., Malina D., Rudnicka K., Michlewska S., Królczyk J.B., Sobczak-Kupiec A. (2021). Measurement methods for the mechanical testing and biocompatibility assessment of polymer-ceramic connective tissue replacements. Meas. J. Int. Meas. Confed..

[19] Al-arjan W.S., Umar M., Khan A., Nazir S. (2020). Pectin/Graphene Oxide/Nano-Hydroxyapatite Based Nanocomposite Scaffolds with Controlled Release of Drug for Bone Tissue Engineering: In-Vitro Evaluation of Biocompatibility and Cytotoxicity. Coatings.

[20] Adamiano A., Iafisco M., Sandri M., Basini M., Arosio P., Canu T., Sitia G., Esposito A., Iannotti V., Ausanio G. (2018). On the use of superparamagnetic hydroxyapatite nanoparticles as an agent for magnetic and nuclear in vivo imaging. Acta Biomater..

[21] Caballé-Serrano J., Zhang S., Sculean A., Staehli A., Bosshardt D.D. (2020). Tissue integration and degradation of a porous collagen-based scaffold used for soft tissue augmentation. Materials.

[22] Lee S., Lee D.S., Choi I., Pham L.B.H., Jang J.H. (2015). Design of an osteoinductive extracellular fibronectin matrix protein for bone tissue engineering. Int. J. Mol. Sci..

[23] Ong J., Zhao J., Justin A.W., Markaki A.E. (2019). Albumin-based hydrogels for regenerative engineering and cell transplantation. Biotechnol. Bioeng..

[24] Kang J.I., Park K.M. (2021). Advances in gelatin-based hydrogels for wound management. J. Mater. Chem. B.

[25] Rogalinski T., Herrmann S., Brunner G. (2005). Production of amino acids from bovine serum albumin by continuous sub-critical water hydrolysis. J. Supercrit. Fluids.

[26] Hong S., Choi D.W., Kim H.N., Park C.G., Lee W., Park H.H. (2020). Protein-based nanoparticles as drug delivery systems. Pharmaceutics.

[27] Aramwit P. (2016). Introduction to Biomaterials for Wound Healing.

[28] Lichtenberg J.Y., Ling Y., Kim S. (2019). Non-specific adsorption reduction methods in biosensing. Sensors.

[29] Kinnari T.J., Peltonen L.I., Kuusela P., Kivilahti J., Könönen M., Jero J. (2005). Bacterial adherence to titanium surface coated with human serum albumin. Otol. Neurotol..

[30] Peltonen L.I., Kinnari T.J., Aarnisalo A.A., Kuusela P., Jero J. (2007). Comparison of bacterial adherence to polylactides, silicone, and titanium. Acta Otolaryngol..

[31] Poralan G.M., Gambe J.E., Alcantara E.M., Vequizo R.M. (2015). X-ray diffraction and infrared spectroscopy analyses on the crystallinity of engineered biological hydroxyapatite for medical application. IOP Conf. Ser. Mater. Sci. Eng..

[32] Wei M., Ruys A.J., Milthorpe B.K., Sorrell C.C. (2005). Precipitation of hydroxyapatite nanoparticles: Effects of precipitation method on electrophoretic deposition. J. Mater. Sci. Mater. Med..

[33] Zhuang Z., Fujimi T.J., Nakamura M., Konishi T., Yoshimura H., Aizawa M. (2013). Development of a,b-plane-oriented hydroxyapatite ceramics as models for living bones and their cell adhesion behavior. Acta Biomater..

[34] Madupalli H., Pavan B., Tecklenburg M.M.J. (2017). Carbonate substitution in the mineral component of bone: Discriminating the structural changes, simultaneously imposed by carbonate in A and B sites of apatite. J. Solid State Chem..

[35] International Organization for Standardization (2008). Implants for Surgery—Hydroxyapatite—Part 1: Ceramic Hydroxyapatite.

[36] Ishigaki Y., Kinoshita T., Nakano K., Yamaguchi K., Shibata N., Aoi K., Nii S., Akita S. (2012). Autonomous stepwise process for adsorption, reduction, and desorption of chromium ions by using Hydrogel beads having poly(ethylene glycol) chains. J. Chem. Eng. Jpn..

[37] Takeshita T., Matsuura Y., Arakawa S., Okamoto M. (2013). Biomineralization of hydroxyapatite on DNA molecules in SBF: Morphological features and computer simulation. Langmuir.

[38] Marques P.A.A.P., Serro A.P., Saramago B.J., Fernandes A.C., Magalhães M.C.F., Correia R.N. (2003). Mineralisation of two phosphate ceramics in HBSS: Role of albumin. Biomaterials.

[39] Matsumoto T., Okazaki M., Inoue M., Hamada Y., Taira M., Takahashi J. (2002). Crystallinity and solubility characteristics of hydroxyapatite adsorbed amino acid. Biomaterials.

[40] Ferraris S., Yamaguchi S., Barbani N., Cazzola M., Cristallini C., Miola M., Vernè E., Spriano S. (2020). Bioactive materials: In vitro investigation of different mechanisms of hydroxyapatite precipitation. Acta Biomater..

[41] Feller L., Chandran R., Khammissa R.A., Meyerov R., Jadwat Y., Bouckaert M., Schechter I., Lemmer J. (2014). Osseointegration: Biological events in relation to characteristics of the implant surface. S. Afr. Dent. J..

[42] Mendonça G., Mendonça D.B.S., Aragão F.J.L., Cooper L.F. (2008). Advancing dental implant surface technology—From micron- to nanotopography. Biomaterials.

[43] Wennerberg A., Albrektsson T. (2009). Effects of titanium surface topography on bone integration: A systematic review. Clin. Oral Implants Res..

